# The World’s Congress Auxiliary of the World’s Columbian Exposition

**Published:** 1892-09

**Authors:** 


### THE WORLD'S CONGRESS AUXILIARY OF THE WORLD'S COLUMBIAN EXPOSITION.

### Department of Medicine—General Division of Dental and Oral Surgery.

PRELIMINARY ADDRESS OF THE COMMITTEE ON A DENTAL CONGRESS.

It is the aim of the World's Columbian Exposition to bring together the evidences of material progress and achievement of the civilization of the world, and so arrange them that every department of human endeavor may be studied and examined through all its various grades of development.

It is also their desire to represent the intellectual and scientific development and achievement of the entire civilized world by a series of great congresses to be held during the progress of the Exposition.

In pursuance of this object the World's Congress Auxiliary was organized by the World's Columbian Exposition, and it has received the recognition and support of the government of the United States.

It is the plan of the World's Congress Auxiliary to bring into communication, through these congresses, the best thinkers and workers in every department of knowledge, including religion, science, philosophy, literature, art, agriculture, trade and labor, etc., and by the presentation and interchange of ideas, methods, theories and practical experiences to promote the advancement of all that is noblest and best of our present civilization.

Committees have therefore been appointed to organize a series of congresses, representing nearly every field of thought, and of speculative and practical endeavor.

In the field of professional achievement, medicine and surgery in their various special applications, will form a very large and interesting feature of the work of the World's Congress Auxiliary.

Dentistry is an important department of medical science and an outgrowth of our modern civilization. Its present perfection is in considerable degree due to the thought and labor of American minds.

The history of modern dentistry is covered by a period of less than two generations, and yet it has advanced from the rude operations practiced by the blacksmiths and barbers, to one of the most scientific and exact of the specialties of the healing art.

Scientific dentistry had its birth in the United States of America. This country has the proud distinction of having organ zed the first school for the teaching of dental science, and the establishment of the first periodical journal devoted to the interests of dentistry, while very many of the most useful appliances and scientific methods have originated on this side of the Atlantic.

It is therefore eminently fitting that dentistry be represented at the World's Columbian Exposition by a display of the progress which has been made in the development of its materials, instruments, appliances, processes and methods of a practical nature, and in scientific research, literature and professional education.

With this end in view the dentists of the United States took steps in August, 1890, to organize such a World's Congress, by the appointment of a General Executive Committee, to whom the whole matter of organizing and conducting the congress was referred.

The work, therefore, of the Committee on Dental Congresses appointed by the World's Congress Auxiliary will be chiefly in co-operation with that General Executive Committee, in publishing to the world from time to time the progress of the work of organization, in promoting the interests of the congress in every way within their power, and keeping it in harmony with the general plans of the World's Congress Auxiliary.

Every effort will be made to secure the best talent in the presentation of scientific subjects and in practical demonstrations.

The World's Columbian Exposition, through its directory, will provide ample accommodations for all the various World's

Congresses to be held in Chicago in 1893. The Memorial Art Palace, now in process of erection upon the shore of Lake Michigan, and located near the center of the city, will be devoted to this purpose. This building will contain two large audience rooms, with a seating capacity of about three thousand each, which will be used for the general congresses of the various departments, besides numerous smaller rooms, suitable for the chapters and sections of the congresses, thus affording for the Dental Congress ample accommodations for clinical demonstrations of a suitable nature.

During the sessions of the Dental Congress several popular evening meetings will be held to which the general public will be invited. At these meetings, which are intended to be educational, illustrated lectures will be delivered by some of the most eminent men of the profession upon topics which are deemed to be of vital importance to the public. These meetings will be especially under the control and management of the World's Congress Auxiliary. When the suggestions of the Advisory Councillors of the Dental Congress shall have been received as to the most interesting and vital questions to be presented, a programme will be arranged for publication.

A cordial invitation is extended to the dentists of the world to take part in the scientific work of the Congress by the presentation of papers and discussions, or demonstrations of new or improved methods and appliances.

America, and Chicago in particular, will have a hearty welcome for all who may come.

An earnest effort was made to bring the meeting of this Congress in close connection with others of the Department of Medicine, but that effort having proved unavailing, arrangements have been effected under which the meeting of the dental profession will be held on, or near, August 17th, and is expected to continue during the week or ten days following. Definite dates and details will be given in the programme.

Communications in reference to the special work of the Congress should be addressed to Dr. A. O. Hunt, Secretary World's Columbian Dental Congress, Iowa City, Iowa, U. S. A.

Communications in reference to the general work of the World's Congress Auxiliary and suggestions from the Advisory Councillors may be addressed to the Chairman of the Committee.

DR. JOHN S. MARSHALL, *Chairman*,
No. 34 Washington Street, Chicago.

DR. A. W. HARLAN, *Vice-Chairman.*

DR. G. V. BLACK,

DR. N. NELSON,

DR. C. N. JOHNSON,

DR. A. E. BALDWIN,

DR. GEORGE H. CUSHING,

DR. A. W. FREEMAN,

DR. E. S. TALBOT,

DR. GEORGE A. CHRISTMAN,

Committee of the World's Congress Auxiliary on a Dental Congress.

---

THE WOMAN'S COMMITTEE OF THE WORLD'S CONGRESS AUXILIARY ON A DENTAL CONGRESS.

DR. HATTIE E. LAWRENCE, *Chairman.*
DR. MARIE T. BACON, *Vice Chairman.*

Dr. Emma Beanham, Dr. Louise Peterson, Dr. Rebecca McIntosh.

WORLD'S CONGRESS HEADQUARTERS, CHICAGO, JUNE, 1892.

---

PARTIAL LIST OF THE
ADVISORY COUNCIL OF THE WORLD'S CONGRESS AUXILIARY ON A DENTAL CONGRESS.

Dr. W. D. Miller, Berlin, Germany.
Dr. F. Busch, Berlin, Germany.
Dr. Thos. W. Evans, Paris, France.
Dr. E. Magitot, Paris France.
Dr. G. W. Sparrock, Lima, Peru.
Dr. W. B. Macleod, Edinburgh.
Dr. A. W. W. Baker, Dublin.
Dr. Earnst Sjoberg, Stockholm, Sweden.
Dr. Charles S. Tomes, London, England.
Dr. W. H. Coffin, London, England.
Dr. W. Geo. Beers, Montreal, Canada.
Dr. H. C. Edwards, Madrid, Spain.
Dr. E. Lecaudy, Paris, France.
Dr. —Plattschick, Pavia, Italy.
Dr. Jos. Arkovy, Buda Pesth, Hungary.
Dr. C. Redard, Geneva, Switzerland.
Dr. J. G. Van Marter, Rome, Italy.
Dr. W. H. Morgan, Nashville, Tenn.
Dr. W. H. Dwinelle, New York City.
Dr. R. B. Winder, Baltimore, Md.
Dr. Elisha G. Tucker, Boston, Mass.
Dr. W. W. H. Thackston, Farmville, Pa.
Dr. J. B. Rich. Washington, D. C.
Dr. J. D. White, Philadelphia, Pa.
Dr. W. H. Eames, St. Louis, Mo.
Dr. J. B. Patrick, Charleston, S. C.
Dr. C. C. Knowles, San Francisco, Cal.
Dr. F. J. S. Gorgas, Baltimore, Md.
Dr. G. V. Black, Jacksonville, Ill.
Dr. J. E. Garretson, Philadelphia, Pa.
Dr. R. Finlay Hunt, Washington, D. C.
Dr. E. Bacon, Portland. Me.
Dr. Benjamin Lord, New York City.
Dr. A. L. Northrop, New York City.
Dr. W. W. Allport, Chicago, Ill.
Dr. W. W. Walker, New York City.
Dr. L. D. Carpenter, Atlanta, Ga.
Dr. J. Y. Crawford, Nashville, Tenn.
Dr. W. J. Barton, Paris, Texas.
Dr. J. Taft, Cincinnati, O.
Dr. C. S. Stockton, Newark, N. J.
Dr. L. D. Shepard, Boston, Mass.
Dr. H. J. McKellops, St. Louis, Mo.
Dr. A. O. Hunt, Iowa City, Iowa.
Dr. H. B. Noble, Washington, D. C.
Dr. Geo. W. McElhaney, Columbus, Ga.
Dr. J. C. Storey, Dallas, Texas.
Dr. M. W. Foster, Baltimore, Md.
Dr. A. W. Harlan, Chicago, Ill.
Dr. J. S. Marshall, Chicago, Ill.

PARTIAL LIST OF THE WOMAN'S ADVISORY COUNCIL ON A DENTAL CONGRESS.

Dr. Lucy Hobbs Taylor, Lawrence, Kan.
Dr. Olga Neymann, New York City.
Dr. Jessie Ritchie, Des Moines, Iowa.
Dr. Annie D. Romborgen, Phila. Pa.
Dr. Jennie Hilton, Fort Atkinson, Wis.
Dr. Clara W. McNaughton, Wash., D. C.
Dr. Kate C. Moody, Mendota, Ill.
Dr. Martha J. Robinson, Cleveland, O.
Dr. Annie F. Reynolds, Boston, Mass.
Dr. Mary T. Benfield, Honolulu, Hawaii.
Dr. Mary Holst, Aarhuus, Denmark.
Dr. Henriette Tiburtius-Hirschfeld, Berlin, Germany.
Dr. Helene Wongl v Swiderska, St. Petersburg, Russia.
Dr. Bella Meller, Vienna, Austria.
Dr. Helene Freudenheim, Konigsberg, Germany.
Dr. Marie M. Schneeyans, Eberfeld, Germany.
Dr. Emma Lacy, London, England.
Dr. Clotilde Lenta, Rome, Italy.

